# Is Job Control a Double-Edged Sword? A Cross-Lagged Panel Study on the Interplay of Quantitative Workload, Emotional Dissonance, and Job Control on Emotional Exhaustion

**DOI:** 10.3390/ijerph14121608

**Published:** 2017-12-20

**Authors:** Anne-Kathrin Konze, Wladislaw Rivkin, Klaus-Helmut Schmidt

**Affiliations:** 1 Leibniz Research Centre for Working Environment and Human Factors at the Technical University Dortmund, 44139 Dortmund, Germany; w.rivkin@aston.ac.uk (W.R.); schmidtkh@ifado.de (K.-H.S.); 2 Work and Organizational Psychology Department, Aston University, Birmingham B4 7ET, UK

**Keywords:** cross-lagged panel, emotional dissonance, emotional exhaustion, job control, job demands-control model, quantitative workload

## Abstract

Previous meta-analytic findings have provided ambiguous evidence on job control as a buffering moderator of the adverse impact of job demands on psychological well-being. To disentangle these mixed findings, we examine the moderating effect of job control on the adverse effects of quantitative workload and emotional dissonance as distinct work-related demands on emotional exhaustion over time. Drawing on the job demands-control model, the limited strength model of self-control, and the matching principle we propose that job control can facilitate coping with work-related demands but at the same time may also require employees’ self-control. Consequently, we argue that job control buffers the adverse effects of quantitative workload while it reinforces the adverse effects of emotional dissonance, which also necessitates self-control. We examine the proposed relations among employees from an energy supplying company (*N* = 139) in a cross-lagged panel study with a six-month time lag. Our results demonstrate a mix of causal and reciprocal effects of job characteristics on emotional exhaustion over time. Furthermore, as suggested, our data provides evidence for contrasting moderating effects of job control. That is, job control buffers the adverse effects of quantitative workload while it reinforces the adverse effects of emotional dissonance on emotional exhaustion.

## 1. Introduction

Over the past decades, work environments have undergone significant changes caused by fast developing technologies, an increasing focus on service orientation and highly competitive conditions. Due to these changes, employees are confronted with an increase in mentally stressful job demands. In 2016, roughly a third of EU employees reported to work to tight deadlines at least half of their time (37% [1]) and to suppress their emotions at work either all or most of the time (31% [1]). Time pressure and high levels of work volume, in other words, high quantitative workload has continuously increased in advanced industrialized societies [2]. Furthermore, due to the increase of service sector occupations, employees increasingly face high emotional demands, as they are required to display emotions and behaviors that are in line with organizational rules or expectations, even if this means “faking” emotions and suppressing true feelings (referred to as *emotional dissonance* [3]). So far, research has provided convincing evidence that high levels of these job demands can cause impairments in employees’ psychological well-being [4,5], such as burnout. A core characteristic of burnout is emotional exhaustion [6], which reflects a lack of energy and adversely affects important organizational outcomes, such as turnover and job performance [7]. Therefore, understanding how burnout develops over time and investigating the interplay between job characteristics in order to identify factors, which may protect employees’ psychological well-being has become of central interest in the field of occupational health psychology [5].

One of the most influential models guiding this research is the Job Demand–Control (JD-C) model [8,9], which identifies two crucial job characteristics that influence employees’ well-being. On the one hand, *job demands* refer to stressors (i.e., having to work under time pressure or having to cope with emotionally demanding situations), which require employees’ effort. On the other hand, *job control* is defined as the extent to which an employee has potential control over decisions concerning when, where, and how to perform work tasks. According to the JD-C model [8,9], impairments in psychological well-being are most likely to arise in jobs with high job demands and low job control.

This theoretical notion has been examined on the basis of two distinct hypotheses [4,5,10,11]: First, the *strain hypothesis* suggests job demands and job control as distinct predictors of well-being, implying that these two job characteristics are directly related to psychological well-being. Second, the *buffer hypothesis* proposes that job control interacts with job demands in predicting well-being. More specifically, job control is argued to buffer the adverse impact of job demands and thus, mitigate their detrimental consequences on employees’ psychological well-being.

Despite its dominant prevalence in occupational health research, empirical evidence for the validity of the JD-C model has been mixed so far. While three meta-analyses found compelling evidence for the strain hypothesis in cross-sectional studies [4,5,11], longitudinal evidence for the additive (direct) effects of job demands and job control on well-being is not yet definite [4,5,11,12]. Moreover, as the majority of studies on the JD-C model have been conducted on the basis of cross-sectional designs, questions concerning the causality of effects still remain. Accordingly, de Lange and colleagues [11] have called for more longitudinal research “to obtain a fuller understanding of the dynamic interplay between work and worker health” by analyzing reversed and reciprocal relations (p. 302).

Making this picture even more complicated, empirical evidence for the buffer hypothesis is even less consistent. Several meta-analyses found support for the interaction of job demands and job control in less than half of the studies (only 30–48% of studies supported the buffer hypothesis [4,5,11]). Moreover, Tanner and colleagues [13] also demonstrate counterintuitive reinforcing effects of job control in the relation between misfit of personal and organizational standards and depressive symptoms. In light of this ambiguous evidence on job control as a buffering moderator, several methodological and conceptual explanations have been proposed, such as small sample size [11], lack of longitudinal studies [4,11], and the use of (overly) general measures for job demands [14,15,16]. However, most of these explanations did not consider theoretical arguments for the lack of evidence on interactions between job demands and job control. One notable exception is a study by de Jonge and colleagues [14], who contend that the failure to distinguish between different, domain-specific types of job demands (i.e., mental demands, emotional demands, and physical demands) may be one reason for the inconsistent findings on the JD-C model. This proposition is consistent with the matching principle [17], which suggests that the buffering effect of resources will most likely emerge, if the type of resource matches the specific demand.

In the present study, we aim to advance this proposition by analyzing quantitative workload and emotional dissonance as two distinct job demands. We argue that compared with quantitative workload, which can require different forms of effort (i.e., cognitive-, self-control- or even motor effort), coping with emotional dissonance predominantly necessitates the exertion of volitional self-control to suppress genuine emotions and express emotions, which are not truly felt [14]. In line with the strength model of self-control, which suggests that self-control draws on and depletes a limited regulatory resource [18,19], and that the chronical depletion of this resource is related to adverse consequences for employees’ psychological well-being (i.e., emotional exhaustion) [19], we propose that the particularly high requirement to exert self-control can account for the adverse consequences of emotional dissonance. In addition, drawing on experimental findings, which show that decision making can also consume this regulatory resource [20], we propose that job control may also require employees’ self-control, because it involves thoughtful decision-making and choosing between different strategies to accomplish work tasks. Integrating these arguments and the matching principle [17], we propose that on the one hand, job control as a resource can provide coping opportunities (i.e., rescheduling tasks) when dealing with quantitative workload and through this match may buffer the adverse consequences of quantitative workload. On the other hand, we argue that job control may not be appropriate when coping with emotional dissonance. More specifically, we propose that job control may even reinforce the adverse effects of emotional dissonance, because both job characteristics are thought to necessitate self-control, resulting in overadditive depletion of the limited regulatory resource and associated emotional exhaustion.

Given the limitations of existing JD-C studies (first, a lack of longitudinal study designs that examine the causality of effects and second, a lack of theoretical reasoning for inconsistent findings on the buffer hypothesis that might be accounted for by a one-sided, biased view on job control), the present study may offer at least two contributions to research on the JD-C model. First, we intend to broaden the understanding of the causality of effects by analyzing the relations in a two-wave cross-lagged panel design, thereby considering potential reversed and reciprocal effects. Second, we integrate notions from the JD-C model [8,9], the limited strength model of self-control [19], and the matching principle [17] to introduce theoretical refinements of the JD-C model. We argue that job control can be a double-edged sword, which can exert beneficial, as well as harmful effects on emotional exhaustion, contingent on the type of demand the employee faces. By that means, we provide a possible explanation for previously inconsistent findings on the buffer hypothesis [13]. 

In the following, we first focus on the strain hypothesis to develop distinct predictions for the direct effects of quantitative workload, emotional dissonance, and job control on emotional exhaustion. After that, we discuss the buffer hypothesis to shed new light on the potential ambiguous role of job control as a moderator. Our predictions are then tested in a two-wave cross-lagged panel study design. 

### 1.1. The Strain Hypothesis: Examining the Causality of Direct Effects

According to the strain hypothesis, high job demands, defined as “psychological stressors involved in accomplishing the workload” ([9], p. 291), are proposed to directly impair psychological well-being. Nowadays, a plethora of studies has provided strong empirical support for this hypothesis [4,5,11].

While the relations between job demands and well-being are widely acknowledged, empirical evidence on the underlying causality of these relations is still rare [11]. This shortcoming is caused by an overrepresentation of cross-sectional studies within the JD-C literature, given that about 80% of studies have been conducted using cross-sectional designs [5,12]. These study designs, however, do not allow for examining reversed or reciprocal relationships [21] and therefore, cannot rule out the possibility of reversed or reciprocal causality (e.g., job demands and well-being might influence each other reciprocally). For example, Zapf and colleagues [21] suggest several different processes that might account for reciprocal effects (i.e., healthier employees might perceive their jobs more positively and might promote positive changes in their work). Accordingly, researchers [5,11] have emphasized the importance of examining the association between job demands and well-being in longitudinal studies, thereby considering reversed and reciprocal causal relationships. 

Among the most studied job demands in the current JD-C literature, quantitative workload stands out as being predominant [4,5]. High time pressure and excessive amounts of work constitute the core aspects of quantitative workload, which has been shown to be positively related to several indicators of impaired psychological well-being, such as emotional exhaustion [22]. Even though some aspects of high workload may necessitate the exertion of self-control (i.e., resist distractions when working under high time pressure; [22]) overall high workload constitutes a job demand, which can be thought to require a variety of different forms of effort, such as cognitive (i.e., memory and attention) and even motor functions (i.e., precise movements and stamina). For example, to fix a broken electricity line in a short amount of time, technicians need to make use of their previous knowledge (i.e., to figure out the problem) and potentially use some form of motor skills to fix the problem. Accordingly, high quantitative workload is proposed to necessitate different forms of effort, which is associated with load reactions (i.e., increase of heart rate, mental fatigue) and if maintained for longer time periods can result in impairments of psychological well-being [23]. Consistently, we propose that the adverse effects of high quantitative workload on emotional exhaustion are likely to rely on different forms of effort. 

Moreover, complementing notions from existing studies (e.g., [4,5,11]), we examine the causality of effects by analysing whether quantitative workload predicts emotional exhaustion over time. Emotional exhaustion, as one of the core dimensions of burnout, is defined as a lack of energy and a sense of emotional resources being consumed fully by work [6,24]. In the present study, we examine emotional exhaustion as an indicator of well-being because (a) it constitutes a dominant indicator of well-being, which was examined in a plethora of studies drawing on the framework of the JD-C model [11] and (b) it has been suggest to result from the chronic depletion of the regulatory resource underlying acts of self-control [25]. Thus, we predict:

**Hypothesis** **1.** Quantitative workload has a positive effect on the development of emotional exhaustion over time.

Since the emergence of the JD-C model in the 1980s, there has been a decline of manufacturing industries and an increase of service-oriented occupations [26,27]. This transition has led to a shift from physical demands to emotional demands [28,29]. For example, regardless of their true emotions, service employees are expected to be friendly and helpful, health service workers are expected to be caring and nurturing, and funeral home directors are expected to be sober [30]. Contrasting this development, the majority of studies on the JD-C model focused on more traditional job demands (i.e., physical effort and quantitative workload), thereby excluding other important job demands (i.e., emotional demands [14,28]). De Jonge and colleagues [14] concluded that this limitation of existing JD-C studies contrasts with the considerable empirical evidence showing that emotional demands are meaningful and unique predictors of impaired psychological well-being.

Indeed, employees are increasingly required to display organizationally mandated emotions, even when they contradict genuinely felt emotions [3,31]. The extent to which employees experience a mismatch between felt and displayed emotions is commonly referred to as emotional dissonance, a demand that is strongly associated with different indicators of impaired psychological well-being [18,32]. These adverse effects of emotional dissonance can be accounted for by the strength model of self-control [19]. According to this model, acts of self-control involve volitionally inhibiting, altering, and overriding automatic or habitual responses (cf., [33]). These effortful internal processes are proposed to require the expenditure of a limited regulatory resource capacity, thereby rendering it less available for subsequent self-control attempts. Hence, previous research has strongly suggested that coping with emotional dissonance puts high demands on volitional self-control [18], because portraying emotions contrary to one’s genuinely felt emotions can be thought to require continual monitoring of organizationally mandated emotions, effortful suppression of genuine emotions, and a continuous modification of the required emotional expression [34]. While the expression of specific emotions at work might contribute to achieving organizational goals, there is compelling evidence that this adaptation of emotions may come at a cost for employees. In particular, there is broad empirical evidence drawing on the strength model of self-control, which suggests that coping with emotional dissonance drains the limited regulatory resource (e.g., [18,19,34]). Consequently, coping with emotional dissonance, as a potential form of self-control, is likely to draw on and deplete a common regulatory resource capacity. Moreover, when employees frequently exert self-control without being able to replenish their depleted resources, psychological well-being is proposed to be considerably impaired [19]. In line with this proposition, emotional dissonance has been found to be related to symptoms of burnout (such as emotional exhaustion) in cross-sectional, as well as in longitudinal studies (e.g., [33]). 

Taken together, coping with emotional dissonance can be expected to drain a limited regulatory resource, resulting in emotional exhaustion. Again, to examine the causality of effects, this relationship is tested longitudinally. Thus, we hypothesize:

**Hypothesis** **2.** Emotional dissonance has a positive effect on the development of emotional exhaustion over time.

Despite the direct effect of high job demands on impaired psychological well-being, the strain hypothesis further suggests that high job control directly relates to increased psychological well-being. According to Karasek [8], this favorable impact of job control results from the opportunity to exercise judgment and the freedom to select the most appropriate skills and ways to complete work tasks. To the extent that an organization permits its employees to set priorities autonomously and to allocate their efforts most appropriately, feelings of efficacy and the ability to cope with the environment are enhanced [8,30]. Furthermore, as human beings generally strive for self-determination rather than being controlled by others [35], the fulfillment of this need is directly associated with psychological well-being.

Some prior research on job control has conceptualized job control in terms of broad and distal measures, thereby combining different facets of control, with aspects such as job complexity, skill discretion, and learning opportunities [15,36,37]. The use of these broad measures might have limited the probability of detecting true effects, as they involve the risk of masking specific effects of job control [15]. Consequently, Wall and colleagues [36] developed a more specific measure of job control that incorporates timing control (as the employee’s opportunity to determine the scheduling of work) and method control (as the employee’s choice on how to perform a work task). This measure of job control has provided better chances for detecting specific effects of job control [15]. For instance, Wall and colleagues [36] found the hypothesized effects of job control using their more specific measure, whereas parallel analyses applying a broader measure did not show equivalent effects.

We shall notice at this point, that as suggested in the title of this study (“Is Job Control a Double-Edged Sword?”), we expect job control to have differential effects on emotional exhaustion. In short, we argue that the freedom of choice that results from high levels of job control can constitute a beneficial or detrimental boundary condition. On the one hand, we propose that high levels of job control enhance feelings of self-efficacy and self-determination. On the other hand, we suggest that dealing with high job control may require effortful decision-making and active initiative that just like other processes of self-control (i.e., coping with emotional dissonance) may deplete a common regulatory resource [20]. Accordingly, we suggest that under specific circumstances, job control can have an adverse impact on emotional exhaustion. In particular, its potential disadvantages (effortful decision-making that depletes a common regulatory resource), may outweigh its potential advantages (enhanced feelings of self-efficacy) in situations when an employee simultaneously faces high levels of job control and high levels of demands that also necessitate self-control. However, although we argue that dealing with high levels of job control may involve effortful decision-making, we do not expect that this internal process on its own is crucial in substantially depleting the regulatory resource. Thus, in accordance with the JD-C model as the dominant theoretical framework of the current study and in line with a plethora of studies on this model, we expect job control to exhibit a direct, beneficial impact on employees’ emotional exhaustion. 

In sum, we suggest that job control (defined as timing and method control) offers the freedom to allocate time and effort in completing work tasks in a way that is most suitable for each individual. Therefore, we expect job control to be negatively related to feelings of emotional exhaustion:

**Hypothesis** **3.** Job control has a negative effect on the development of emotional exhaustion over time.

Because we test Hypotheses 1 to 3 in a cross-lagged panel design, we hypothesize that quantitative workload, emotional dissonance, and job control as distinct job characteristics at Time 1 (T1) predict changes in emotional exhaustion from T1 to Time 2 (T2), while simultaneously considering potential reversed or reciprocal effects. By choosing this method of analysis, we intend to examine the direction of the relations between these job characteristics and emotional exhaustion.

### 1.2. The Buffer Hypothesis: Is Job Control a Double-Edged Sword?

In addition to the direct effects that are postulated in the strain hypothesis, the JD-C model further suggests that impairments in psychological well-being can also result from *joint effects* of job demands and job control [8]. In particular, the buffer hypothesis proposes that high levels of job control can protect employees from the adverse consequences of high job demands [8]. This moderating effect of job control is expected to result from the opportunity to decide how to perform work tasks, the freedom to decide how to schedule and pace the work processes and the choice over methods to accomplish work goals [9]. From an action-regulation perspective, job control offers the possibility to appraise and seek constructive coping responses by addressing job demands at times and in ways that fit an employee’s coping strategy [38]. If employees are granted the freedom to exercise control over their tasks, methods, scheduling, and pacing, they have the possibility to face demands when they are best able to do so and in ways they find most appropriate [15]. Consequently, employees with high levels of control over their work are able to utilize a wide range of strategies when being confronted with high job demands [39], such as deciding on the pacing of their work and taking short breaks [40], prioritizing goals [41], or choosing between different methods to accomplish tasks [42].

It is especially this hypothesis that has received much attention, because its central implication is that job demands can be increased with little or even no threat to employees’ psychological well-being, as long as there is sufficiently high job control [36]. However, it is also this hypothesis that has received limited empirical support. For example, in a review of JD-C studies conducted between 1998 and 2007, Häusser and colleagues [5] found support for the buffer hypothesis only in 29 out of 97 tests (30%). Besides the low support rates in cross-sectional studies, longitudinal support for the lagged interaction effect was even weaker (10% [5]). Moreover, a longitudinal study by Tanner and colleagues [13] has even demonstrated that job control can reinforce the adverse effects of misfit between organizational values and personal practices as a job related stressor.

Due to this inconclusive evidence on the buffer hypothesis, van der Doef and Maes [4] further examined existing JD-C studies and detected that non-supportive findings were most likely when job demands and job control were broadly conceptualized [4]. On the other hand, supportive findings were more likely when more focused measures were applied and when the type of control corresponded to the type of demand. In other words, job control only buffers the adverse consequences of high job demands, if the type of job control is applicable to the type of demand. This proposition is in line with the matching principle, which suggests that the match of demands and protective resources, such as job control increases the probability of demonstrating interactive effects on well-being [17].

Consistently, in the present study, we propose that employees, who face high quantitative workload and thus, feel pressured by challenging deadlines and excessive amounts of work, will benefit from high job control. More specifically, when confronted with time pressure, timing control as a matching resource offers the possibility to adjust the scheduling of work. Similarly, when facing excessive workloads, method control offers the opportunity to choose how to perform the task. Therefore, when facing high quantitative workload, employees with high levels of job control can control how they conduct their work tasks, they can decide on the pacing and timing, and they can choose the methods involved in how to accomplish their work-related goals [42]. Consequently, when confronted with high quantitative workload, employees with high levels of job control modify their work processes in order to cope with the workload in a way that fits their coping strategy. Therefore, they will feel less exhausted than employees with low levels of job control, who face the same amount of quantitative workload. Hence, we hypothesize:

**Hypothesis** **4.** Job control moderates (i.e., buffers) the positive impact of quantitative workload on the development of emotional exhaustion: The relation is attenuated as a function of job control.

Expanding the approach that job control only buffers the adverse consequences of high job demands, if the type of control is applicable to the type of demand [4,13], we believe that job control (as timing and method control) will not buffer the impact of emotional dissonance. Given that many sources of emotional dissonance, such as customer complaints or angry clients, occur unexpectedly, coping with these emotional demanding situations cannot be planned and scheduled proactively. In contrast, employees are required to deal with these situations in the moment they occur. Therefore, job control, as the opportunity to determine the scheduling of work, will not be beneficial when coping with emotional dissonance because it does not appropriately match this demand [17]. Accordingly, Grandey and colleagues [43] concluded that job control does not impact the need to control tempers or to trigger positive feelings in others, because even the most autonomous employees (i.e., managers) need to regulate emotions. Thus, we do not expect job control to buffer the adverse consequences of coping with emotional dissonance on psychological well-being. 

On the contrary, we propose that high job control in combination with high emotional dissonance may even intensify the adverse effects on psychological well-being. This assumption is derived by drawing from research on decision-making and self-regulation. While job control offers several favorable opportunities, it is also likely to require thoughtful decision-making. The freedom to decide when and how to accomplish work tasks and the expectation to choose wisely between different options and strategies can render psychological costs:

“*It leaves people indecisive about what to do and why. Freedom of choice is a two-edged sword, for just on the other side of liberation sits chaos and paralysis*.”

—Schwartz, in *The Tyranny of Freedom* ([44], p. 87)

In several studies, Vohs and colleagues [20] demonstrated that making choices is depleting and that this depletion results from thinking about and comparing different options. Employees with high levels of job control are expected to organize their work process autonomously, thereby flexibly adapting their behavior according to changing time schedules or organizational rules. These expectations cannot be fulfilled by rigid, automatic, or habitual behavioral patterns; rather, they seem to cause employees to constantly compare different behavioral options and to identify the best strategy to accomplish the work task. This contemplation of alternatives and the selection among them is an effortful internal act that requires more than habitual behavioral patterns [20]. 

Instead, decision-making is likely to involve the self’s executive function, which initiates and maintains action and regulates the self. This view of decision-making as an executive control process implies that choosing between different strategies to accomplish a work task may draw on and deplete a common, limited regulatory resource [45]. When choosing between different strategies, a decision maker must trade off advantages and disadvantages, which are thought to induce costly cognitive conflicts [46]. In fact, several studies have demonstrated that decision making, just like other self-control processes, consumes a limited regulatory resource [19], thereby rendering the resource less available for further acts of self-control [20]. Accordingly, the chronic depletion of this resource is likely to manifest in high levels of impaired psychological well-being [19].

Hence, if coping with high emotional dissonance is likely to consume a limited regulatory resource and if dealing with high job control may also consume the same limited regulatory resource, in line with the strength model of self-control, we expect that these two job characteristics will exert interactive effects on employees’ psychological well-being. In other words, because both job characteristics seem to draw on and deplete a common regulatory resource, we propose that the positive relation between emotional dissonance and emotional exhaustion is likely to be amplified as a function of job control. Strong empirical support for this argument is provided by Diestel and Schmidt [47], who demonstrated that simultaneous coping with two distinct demands on self-control causes higher levels of strain than accounted for by the additive effects of each demand. Thus, in jobs that are characterized by high levels of emotional dissonance and high levels of job control, employees may have to exert higher amounts of self-control, which is expected to draw on the common limited resource, resulting in disproportionate levels of regulatory resource depletion. Consequently, depletion of this resource is more likely and recovery of that resource is more difficult compared to coping with only one of these job characteristic. Therefore, we hypothesize:

**Hypothesis** **5.** Job control moderates (i.e., reinforces) the positive impact of emotional dissonance on the development of emotional exhaustion: The relation is reinforced as a function of job control.

## 2. Methods

### 2.1. Research Design and Participants

A complete two-wave panel survey was conducted among employees from an energy supplying company in Germany. In this sample, quantitative workload (many deadlines and high workload due to competitive pressure) and emotional dissonance (customer interactions, especially customer complaints) constitute major stressors. The study was announced at staff meetings and via emails sent by managers and the research team. Invitations to the online surveys were sent to employees’ email addresses who completed the surveys during working hours. Participation was anonymous, voluntary and not motivated by any incentives. Moreover, the study was conducted with agreement of the workers’ council. The self-report surveys were sent on two occasions with a six-month time lag. By choosing this time lag, we complied with a recent request for longitudinal studies with time lags shorter than one year [11]. As de Lange and colleagues [11] pointed out, the majority of longitudinal JD-C studies have collected data at least one year apart. Hence, in this study, we adopted the approach by Baillien and associates [48] and chose a six-month time lag to enhance insights in shorter term consequences of job characteristics and job control.

At the first occasion (in July/August 2016), 238 out of 452 employees followed the invitation to participate in the study (response rate 52.7%). At the second measurement point (in January/February 2017), 195 employees took part (response rate 43.1%); leading to an overlap of 139 participants who responded to the surveys on both occasions. Of these participants, 34.5% were female, 16.2% worked part-time, and the majority of participants (62.0%) were at least 46 years old. On average, employees had been working in the company for 20.0 years (*SD* = 10.7) at the first measurement point.

### 2.2. Measures and Control Variables

We designed the study as a complete two-wave cross-lagged panel. Therefore, questionnaires at both occasions were identical and comprised of the same scales.

*Quantitative workload* was measured with three items that were based on the *Short Questionnaire for Job Analysis* by Prümber and colleagues [49]. The original two-item subscale of quantitative demands was extended by constructing a third item [22]. Overall, the measure addressed demanding aspects, such as time pressure and work volume and thus, covered demands that have been considered in prior investigations of the JD-C model [4,15,50]. An exemplary item is “At my work, I often feel that I am pressed for time.” Participants responded on a five-point scale ranging from 1 (*strongly disagree*) to 5 (*strongly agree*).

*Emotional dissonance* was assessed with five items from the *Frankfurt Emotion Work Scale* [51]. Participants were asked to report the frequency of experienced discrepancies between felt emotions and those required by the job role (e.g., “How often do you have to show feelings at work that you do not really feel?”). The items were slightly modified by asking specifically about interactions with colleagues and customers. The five-point response format ran from 1 (*never*) to 5 (*very often*).

*Job control* was measured by combining items from the timing control (three items) and method control (four items) subscales developed by Jackson and colleagues [52]. This procedure is consistent with previous work on the JD-C model [15,37]. All items, such as “Do you decide on the order in which you do things?” and “Can you choose the methods to use in carrying out your work?” were scored on a five-point scale with anchors from 1 (*not at all*) to 5 (*a great deal*).

*Emotional Exhaustion* as the focal dimension of burnout was assessed with eight items from the German translation [53] of the *Maslach Burnout Inventory* [6]. This specific dimension of burnout refers to feelings of being emotionally overextended of emotional and physical resources resulting from demands of one’s work. An exemplary item is “I feel emotionally drained by my work”. Participants responded on a six-point scale that ranged from 1 (*never*) to 6 (*very often*).

All measures revealed satisfactory internal consistencies, as presented in Table 1.

### 2.3. Analytical Procedure

**Figure A1:**
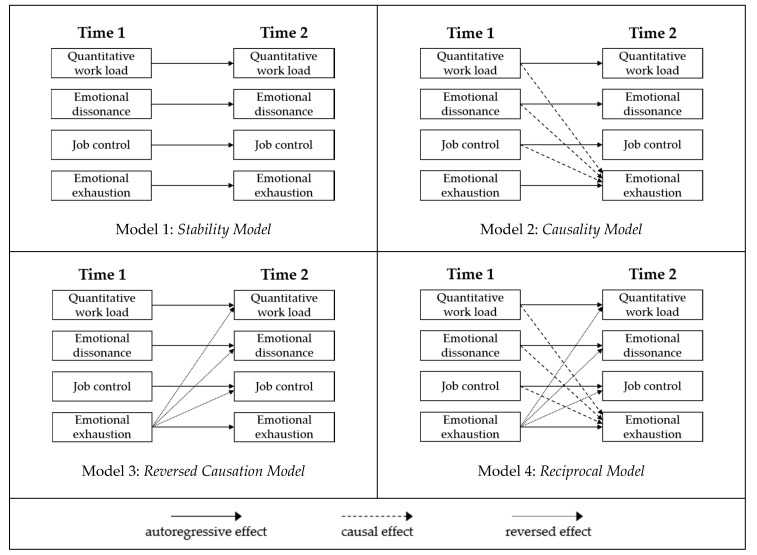
Path models for investigating cross-lagged relationships as suggested by Hakanen and colleagues [55].

To investigate the hypotheses in the cross-lagged panel, we analyzed path models using the software R and the lavaan package [54]. Thereby, we adopted the procedure proposed by Hakanen and colleagues [55]. Accordingly, to gain a deeper understanding of the causal process, we tested several competing full-panel path models to examine the cross-lagged relations . All path models included all study variables at both occasions. Model 1, the *stability model*, only included autoregressive effects in order to control for baseline levels of each variable. By adding the autoregressive effects to the path models, it is possible (a) to examine the stability of the study variables over time, and, more importantly, (b) to predict the change in emotional exhaustion over time. In Model 2, the *causality model*, we added the hypothesized causal relations to the autoregressive effects. Model 3, the *reversed causation model*, combined the autoregressive effects with reversed effects to test the possibility that the effects run opposite to the hypothesized effects. In Model 4, the *reciprocal model*, we combined the causality and the reversed causation model to test whether job characteristics and emotional exhaustion mutually influence each other over time. An illustration of these four competing models is presented in Figure A1 (Appendix A).

We compared these four models on the basis of Comparative Fit Index (CFI ≥ 0.95), Tucker-Lewis Index (TLI ≥ 0.95), Root Mean Square Error of Approximation (RMSEA ≤ 0.05), and *χ*^2^-difference test to identify the best fitting model (numbers in brackets represent cutoff values for good model fit, [56]).

After identifying the best fitting model, the moderation hypotheses (Hypotheses 4 and 5) were tested. Therefore, the interaction terms of (1) quantitative workload and job control and of (2) emotional dissonance and job control were computed by multiplying the z-standardized variables measured at T1. Subsequently, in Model 5, the *moderation model*, these interaction terms were added to the best fitting panel model.

## 3. Results

### 3.1. Analysis of Dropout Effects

To examine if a systematic drop out of respondents between the first and the second occasion might have distorted the results, we compared the panel group (participants who responded at both occasions) with the dropouts (participants who only responded at the first occasion) by calculating *t*-tests with data obtained at T1. There were no significant differences between these two groups, neither for the demographic variables, nor for the study variables. Therefore, we can conclude that no systematic dropout influenced our results.

### 3.2. Measurement Model

As a next step in our analyses, we evaluated the overall measurement model through confirmatory factor analysis (CFA). CFA is a multivariate statistical procedure, which is applied to determine, whether the theoretical factor structure of the constructs is adequately represented by empirical data [57]. In line with the prerequisite of measurement invariance, factor loadings and intercepts were hold equal across time. Moreover, the residual errors of the same items were allowed to be correlated over time. Because emotional exhaustion and job control were each measured with a large number of items, we aggregated the items of both variables into parcels. This procedure offers a number of advantages, such as a reduced number of parameters, more normally-distributed and more reliable measures, and more efficient parameter estimates [58]. For emotional exhaustion, we applied the random parceling method to create two distinct parcels with four items each. For job control, as a multidimensional construct, we applied the domain-representative parceling method by joining items from each subscale to create one parcel for timing control (three items) and one parcel for method control (four items) [59]. The CFA model that allowed all factors to be correlated over time showed a good fit to the data (χ^2^_(221)_ = 327.02, *p* < 0.01; *CFI* = 0.961; *TLI* = 0.952; *RMSEA* = 0.059).

### 3.3. Descriptive Analyses

For an initial overview of the data, descriptive statistics, internal consistencies (Cronbach’s alpha), and correlations among the study variables are presented in Table 1. All correlations between T1 job characteristics and T2 emotional exhaustion were in the hypothesized direction and significant. That is, quantitative workload and emotional dissonance at T1 were positively correlated with emotional exhaustion at T2, while job control at T1 was negatively correlated with emotional exhaustion at T2. 

### 3.4. The Strain Hypothesis: Examining the Causality of Direct Effects

Hypotheses 1–3 focused on the causality of effects between job characteristics and emotional exhaustion. Because the size of our sample did not allow to conduct structure equation modelling with latent constructs [60], we tested our hypotheses on the basis of the means of the relevant constructs and specified cross-lagged path models (all job characteristics and emotional exhaustion at both occasions). In comparison to traditional linear regression analysis, path modelling allows to assess how well different types of theoretical models fit the empirical data [61]. As can be seen in Table 2, all path models showed an acceptable fit to the data, while the stability model obtained the worst fit. The causality model provided a better fit to the data than the stability model (Δ*χ*^2^ = 15.96, *p* < 0.01). Furthermore, the reversed causation model fitted the data better than the stability model (Δ*χ*^2^ = 8.62, *p* < 0.05), but performed worse than the causality model. In comparison with the causality model, the reciprocal model provided no increase in data fit (Δ*χ*^2^ = 7.29, *n.s.*). Thus, our results reveal that the causality model fits the data best. 

Accordingly, Hypotheses 1–3 were tested within the causality model. Parameter estimates of this model are displayed in Table 3. As to be seen, all variables showed significant autoregressive effects, indicating that the variables were somewhat stable with medium (job control) to large effect sizes.

Hypothesis 1 suggested a positive impact of quantitative workload on the change of emotional exhaustion. However, there was no significant lagged effect from quantitative workload to emotional exhaustion (Model 2: *γ* = 0.07; *n.s.*). Therefore, the data does not support Hypothesis 1. This seems especially surprising given the significant bivariate correlation between these two variables (*r* = 0.32, *p* < 0.01).

Hypothesis 2 proposed a positive impact of emotional dissonance on the change of emotional exhaustion over time. The results support this hypothesis, showing that emotional dissonance at T1 had a positive effect on the change of emotional exhaustion from T1 to T2 (Model 2: *γ* = 0.18, *p* < 0.01). 

With regard to Hypothesis 3, we expected a negative impact of job control on the change of emotional exhaustion. Surprisingly, the results reveal a *positive* cross-lagged relationship between job control at T1 and emotional exhaustion at T2 (Model 2: *γ* = 0.14, *p* < 0.01). Thus, Hypothesis 3 was also not confirmed.

### 3.5. The Buffer Hypothesis: Testing Differential Moderating Effects of Job Control

**Figure 1:**
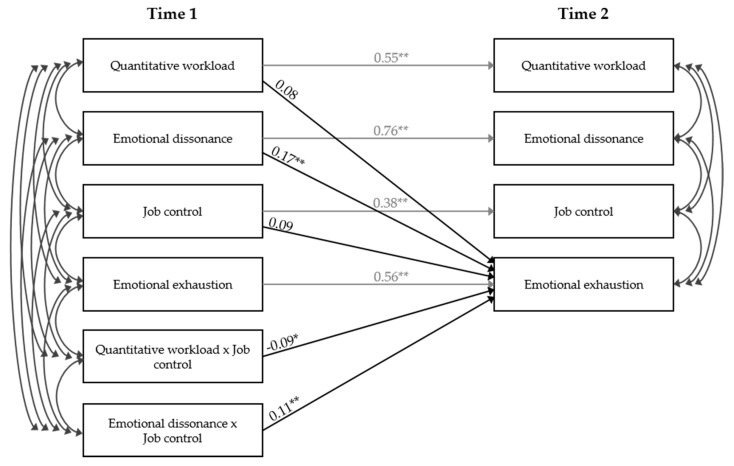
Parameter estimates of Model 5 (moderation model); black paths represent causation paths, grey paths represent autoregressive effects; * *p* < 0.05; ** *p* < 0.01.

Hypothesis 4 and 5 proposed moderation effects of job control. Therefore, the interaction terms of (1) quantitative workload and job control and of (2) emotional dissonance and job control were added to the causality model (Model 5). Results of this final model are displayed in Table 3 and illustrated in Figure 1. Again, this model fitted the data well (*χ*^2^_(12)_ = 11.92, *n.s.*; *CFI* = 1.000; *TLI* = 1.000; *RMSEA* = 0.000).

Hypothesis 4 suggested that job control moderates (buffers) the adverse impact of quantitative workload on the development of emotional exhaustion. In line with this hypothesis, the results reveal that the interaction of job control and quantitative workload was negatively related to the change of emotional exhaustion over time (Model 5: *γ* = −0.09, *p* < 0.05), thereby providing first support for Hypothesis 4.

Furthermore, Hypothesis 5 proposed that job control moderates (reinforces) the adverse effect of emotional dissonance on the development of emotional exhaustion. The results also support this hypothesis, as indicated by the positive interaction effect of job control and emotional dissonance on the change of emotional exhaustion (Model 5: *γ* = 0.11, *p* < 0.01).

In order to facilitate the interpretation of the interaction effects, we plotted the effects and performed simple slope tests as recommended by Preacher and colleagues [62]. Before interpreting the plots, a special aspect of this analysis shall be noticed: Due to the inclusion of autoregressive effects within the path models, baseline levels of emotional exhaustion at T1 were controlled. Consequently, the present model does not predict mean levels in emotional exhaustion at T2, but inter-individual differences in changes of emotional exhaustion from T1 to T2. This procedure enables us to examine how job characteristics affect the development of emotional exhaustion over time.

**Figure 2:**
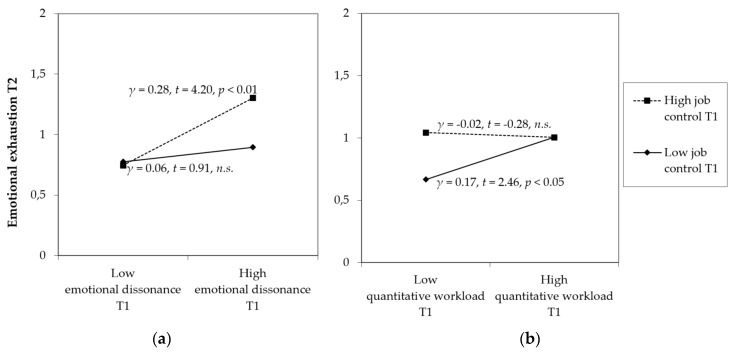
Interaction effects of job control and (**a**) quantitative workload and (**b**) emotional dissonance on emotional exhaustion; high and low values were operationalized by one standard deviation above and below the mean.

As shown in Figure 2, both interactions correspond with Hypotheses 4 and 5. In particular, the buffering effect of job control on the relation between quantitative demands and the development of emotional exhaustion is displayed in Figure 2a. As to be seen, under the condition of high job control, increases in quantitative workload do not affect the change in emotional exhaustion (*γ* = −0.02, *n.s.*). In other words, the change of emotional exhaustion from T1 to T2 does not differ between employees with low vs. high quantitative workload, as long as job control is high. However, if job control is low, increases in quantitative workload adversely affect the development of emotional exhaustion (*γ* = 0.17, *p* < 0.05). Thus, our data fully supports Hypothesis 4. Furthermore, our results also support Hypothesis 5, proposing that job control reinforces the adverse effect of emotional dissonance on the development of emotional exhaustion. As presented in Figure 2b, under the condition of high job control, increases in emotional dissonance adversely relate to the development of emotional exhaustion over time (*γ* = 0.28, *p* < 0.01). Conversely, if job control is low, increases in emotional dissonance do not relate to the development of emotional exhaustion (*γ* = 0.06, *n.s.*). Put differently, the growth in emotional exhaustion from T1 to T2 is subjective to the level of emotional dissonance, only for employees with high job control. Taken together, high levels of job control buffer the adverse effect of quantitative workload, while they reinforce the adverse effect of emotional dissonance on emotional exhaustion over time.

### 3.6. Additional Analyses

In addition to the causal model, we examined the relations of T1 emotional exhaustion to all three job characteristics in the reciprocal model since it has also provided an acceptable data fit. These results indicate that there was only a significant relation between T1 emotional exhaustion and T2 emotional dissonance (*γ* = 0.13, *p* < 0.05), thereby demonstrating a reciprocal rather than a causal relation between emotional dissonance and emotional exhaustion. However, T2 quantitative workload and T2 job control were not affected by T1 emotional exhaustion.

Moreover, due to the fact that our results regarding the strain hypothesis of the JD-C model, specifically (a) the non-significant longitudinal effect of quantitative demands on the change in emotional exhaustion and (b) the significant *positive* longitudinal effect of job control on the development of emotional exhaustion, seem to be incompatible with the otherwise compelling evidence of the strain hypothesis in cross-sectional studies, we decided to conduct additional analyses. In these analyses, we tested the relationships between job characteristics and emotional exhaustion as predicted in the strain hypothesis of the JD-C model in a cross-sectional design. Therefore, we examined path models with data obtained separately at T1 and T2. When the three job characteristics at T1 simultaneously predicted emotional exhaustion at T1, the results revealed the hypothesized pattern for quantitative workload (*γ* = 0.25, *p* < 0.01) and for emotional dissonance (*γ* = 0.33, *p* < 0.01). Moreover, in a cross-sectional snapshot, job control was negatively related to emotional exhaustion (*γ* = −0.22, *p* < 0.01). Accordingly, cross-sectional analyses of T2 revealed that quantitative workload (*γ* = 0.41, *p* < 0.01) and emotional dissonance (*γ* = 0.44, *p* < 0.01) were also significantly and positively related to emotional exhaustion. However, we did not find a significant effect for job control (*γ* = −0.07, *n.s.*) even though the direction of this effect was in line with the strain hypothesis.

In sum our results suggest that although quantitative workload and emotional exhaustion are positively associated in a momentary snapshot, quantitative workload does not predict the change in emotional exhaustion over time. Additionally, if analyzed in a cross-sectional design, job control is negatively associated with emotional exhaustion, while it is positively associated with the development of emotional exhaustion, if analyzed longitudinally. A possible explanation for these findings will be discussed in the following.

## 4. Discussion

In the present study, we investigated the JD-C model longitudinally, thereby analyzing the development of emotional exhaustion over time within a two-wave cross-lagged panel design. Thus, we addressed several limitations of research on the JD-C model by (a) shedding light on the causality of effects through analyzing causal, reversed, and reciprocal path models and by (b) integrating notions from the JD-C model [8,9] and the limited strength model of self-control [19] to introduce theoretical refinements of the JD-C model. By doing so, the present study is one of the first to demonstrate that there is a dark side of job control.

### 4.1. Summary of Results

The results of our study with employees from a German energy supplying company reveal that (1) the causality model provides the best fit to the data of the cross-lagged panel; (2) quantitative workload does not impact the development of emotional exhaustion over time; (3) emotional dissonance has a positive reciprocal relation with emotional exhaustion over time; (4) job control is positively associated with the change in emotional exhaustion; (5) job control buffers the adverse effect of quantitative workload; and, finally, (6) job control reinforces the adverse effect of emotional dissonance on emotional exhaustion. Thus, the results provide strong support for Hypothesis 2, 4, and 5, while Hypothesis 1 and 3 did not receive support. Therefore, the non-significant effect of quantitative workload and the positive effect of job control require closer examination. Given the fact that the direction of the effect of quantitative workload is consistent with Hypothesis 1, the non-significant findings might be attributable to the relatively small sample size of our study (as discussed in the *limitations section*). While this methodological shortcoming might have affected the results, a further explanation can be derived by integrating all findings into a common framework. When investigating the pattern of results in more depth, our findings indicate that when examined concurrently at T1, high levels of quantitative workload are positively related to emotional exhaustion (as indicated by the additional analyses). But, this level remains stable (and will not increase further over time), as long as the employee has sufficient job control to deal with his or her workload. Thus, in line with the buffer hypothesis, only employees with low levels of job control report a positive impact of quantitative workload on the increase in emotional exhaustion. However, over all, this direct effect of quantitative workload is non-significant. Furthermore, in light of the differential moderating effects of job control, an explanation for the positive direct effect of job control on emotional exhaustion can be derived. In particular, with regard to emotional dissonance, the results reveal that coping with emotional dissonance renders psychological costs that become manifest in higher mean levels of emotional exhaustion, as well as in an increase in emotional exhaustion over time. Furthermore, when simultaneously dealing with high levels of job control, the adverse effect of emotional dissonance on the increase in emotional exhaustion is even amplified. These findings seem to support the notion that coping with emotional dissonance and job control both draw on and deplete a common regulatory resource [15]. Accordingly, it can be concluded that whether job control is beneficial or harmful for an employee in the longer run depends on the type of job demand, the employee faces. Overall, the differential interaction patterns between job demands and job control and the unexpected positive direct effect of job control confirm the assumption that job control can exert beneficial, as well as detrimental effects on employees’ psychological well-being.

### 4.2. Theoretical Contributions

Our research offers at least two important theoretical contributions to existing knowledge on the JD-C model and contributes to our understanding regarding the process of how emotional exhaustion as the core dimension of burnout evolves over time. First, the present study sheds light on the underlying causal relations of job characteristics and emotional exhaustion by demonstrating that the causal model provides the best data fit. Therefore, given the fact that only few JD-C studies have applied longitudinal study designs [4,5,11,13], this research extents prior knowledge on the temporal order of variables by applying a longitudinal study design. In particular, low support rates in those few studies that were conducted longitudinally have evoked the proposition that unknown reciprocal or reversed causations might account for part of the associations between job characteristics and psychological well-being, thereby potentially distorting prior findings [21]. Accordingly, the results of the present study are in line with this rather complex picture. While for quantitative demands the relations seem to be non-significant in both directions our data suggests a reciprocal relation between emotional dissonance and emotional exhaustion. Finally, job control has a counterintuitive positive causal effect on emotional exhaustion. Accordingly, our results suggest that the direction of the relation between job characteristics and emotional exhaustion may not be universal for all but unique for individual job characteristics.

Second, given that previous research has provided somewhat inconclusive results on the buffering effect of job control and in light of the ongoing debate on the buffer hypothesis (e.g., [13,58]), our study offers an explanation for the mixed findings. By theoretically integrating notions based on the JD-C model [8], propositions derived from the strength model of self-control [19], and the matching principle [17], our results suggest that job control can be a double-edged sword. On the one hand, the results reveal that job control (as timing and method control) attenuates the adverse effect of workload, thus supporting the idea that job control buffers the adverse consequences of job demands when the type of control is applicable to the type of demand. On the other hand, the results demonstrate that job control intensifies the adverse consequence of emotional dissonance, thereby supporting the proposition that simultaneous coping with high job control and high emotional dissonance is likely to draw on and deplete the same limited regulatory resource. While this result is in line with prior empirical findings indicating (a) that decision-making (i.e., choosing between different strategies to accomplish a work task) involves the self’s executive function and requires expenditure of regulatory resources [20,46], and (b) that resources are most helpful if they match the specific type of job demand [17], it further points out that previous research on job control has largely adopted a one-sided point of view by overlooking the fact that coping with job control can also be effortful. Furthermore, our theoretical proposition may also account for the reinforcing effect of job control in the relation between the misfit of personal and organizational standards and depressive symptoms [13]. Drawing on previous findings that goal incongruence constitutes an organizational boundary condition, which necessitates the exertion of self-control and therefore reinforces the adverse effects of self-control demands on well-being [63], one can assume that misfit between personal and organizational standards may reflect a form of goal incongruence and therefore also require the exertion of self-control. Accordingly, the reinforcing effect of job control on the adverse relations between misfit of organizational and personal standards and depressive symptoms, may substantiate the proposition that self-control and associated depletion of the limited regulatory resource may be an underlying mechanism for the adverse effects of job control. Accordingly, these differential findings on job control contribute to the ongoing debate on the buffer hypothesis by providing evidence for the proposition that whether job control is beneficial or harmful for an employee depends on the type of job demand the employee faces. Therefore, the present study advances our understanding of the JD-C model by identifying a new boundary condition (high emotional dissonance) that has not been addressed in the initial conceptualization of the model. Thus, we contribute to a line of research that has uncovered distinct boundary conditions that need to be considered with regard to the JD-C model, such as personal factors (e.g., self-determination [64]; intrinsic work motivation [65]), cultural aspects (e.g., emotional culture [44]), the optimal level of job control (e.g., curvilinear effects [66]), and the employee’s perception of demands (e.g., hindrance vs. challenge [67]). Supplementing these findings, we add an additional perspective by integrating the JD-C model and the limited strength model of self-control and by stressing the importance of considering whether the employee faces job demands that necessitate considerable self-control efforts.

Taken together, the current study demonstrates that job control is simultaneously associated with beneficial and harmful consequences for employees’ psychological well-being. More specifically, we point out that the direction of the moderating effect of job control depends on the type of demand an employee has to cope with. In line with Schwartz’ [45] argument, our findings support the idea that “there is a dark side to all this freedom from constraint, to all this emphasis on individuals as the makers of their own world” (p. 87). Job control is a double-edged sword: Deciding on the right amount of job control in a specific job requires weighing the advantages of creating feelings of self-determination and self-efficacy against the disadvantages of creating feelings of indecisiveness by the amount of choices.

### 4.3. Limitations and Suggestions for Further Research

Despite its contributions, the present study has also some limitations that need to be discussed and that should be addressed in further research. First, all study variables were assessed by self-reports, thereby increasing the risk that common method variance might have contaminated the observed relations [68]. However, the differential moderation effects of job control are unlikely to be attributable to common method variance and the longitudinal design chosen in the current study further diminishes this risk. Thus, the effects of the current study can claim to reflect valid relations rather than common method artifacts [69]. Nevertheless, future research could enhance the explanatory power of the findings by including more objective measures, such as absenteeism (e.g., [45]).

Second, our data was collected with a discrete time lag of six month. However, as we expect that the development of emotional exhaustion is a continuous process that evolves over time, we cannot be sure that the chosen time lag matches the pivotal period within emotional exhaustion evolves. Consequently, there is a chance that the chosen time lag might not have matched the causal lag [21], which in turn might have distorted our findings. Accordingly, a recommendation for future research is to conduct more longitudinal multi-wave studies with different time lags [14,21].

Third, while the present study provides first evidence for the notion that job control, commonly operationalized as timing and method control, intensifies the adverse consequences of coping with emotional dissonance, further research should uncover whether other types of job control might help employees to cope with emotional dissonance. For this purpose, job control should also be studied in terms of autonomy to determine how, when, and which emotions need to be displayed in the organizational context. In line with the suggestions that job control buffers the impact of job demands only when the type of control is applicable to the type of demand [4], we suggest that control over types of emotions might prevent the adverse effects of emotional dissonance. Thus, research on the interplay between emotional dissonance and control over required emotions could provide new insights that could be used for the development of more specific field interventions.

Fourth, several methodical shortcomings may limit the generalizability of the present results. The sample size was relatively small, thereby reducing the chance to detect significant relations. For example, the effect of quantitative workload on emotional exhaustion over time might be significant in a larger sample. Furthermore, the sample comprised of employees from a single company with some demographical particularities (i.e., relatively high mean age and long tenure). Therefore, further studies in differential occupational settings are needed to increase the generalizability of the arguments provided in the current study.

Fifth, even though the limited strength model of self-control constitutes a dominant theoretical underpinning of our study, we did not measure depletion of the regulatory resource associated with the exertion of self-control. While previous research strongly suggest that the chronic depletion of the limited regulatory resource constitutes an underlying mechanism of the relation between emotional dissonance and emotional exhaustion [25], further research could benefit by examining state self-control as an underlying mechanism of the differential moderating effects of job control in the relation between workload and emotional dissonance to emotional exhaustion.

Finally, yet importantly, we would like to encourage researchers to re-analyse past meta-analytic findings on the JD-C model on the basis of the propositions derived in the current study. More specifically, we suggest that the distinction between demands that necessitate self-control (e.g., emotional dissonance, impulse control, resisting temptations, overcoming inner resistances) and those demands that do not primarily incorporate self-control (e.g., quantitative workload, work overload, time pressure) might account for some of the inconsistent findings on the buffer hypothesis. We believe that researchers and managers alike would benefit from a more refined comprehension of the moderating effect of job control. While the present study might be considered as a first step toward a deeper understanding, taking up past meta-analyses to incorporate self-control as a boundary condition could provide further insights for occupational health research and management.

### 4.4. Practical Implications

Due to the increasing competitive pressure in industrialized countries and the growth of the service sector, quantitative workload and emotional dissonance are expected to increase in the future [2,3]. Thus, the current study offers several important implications for managers and organizations on how to improve employees’ psychological well-being at times that are characterized by increasing amounts of mentally stressful job demands. First and foremost, findings of this study substantiate the importance of conducting job analyses in order to identify the most salient job demand of each employee. Only if the predominant job demand has been classified (i.e., as quantitative demand or as emotional demand), managers should decide on the level of job control. That is, on the one hand, it is beneficial to enhance employees’ job control in jobs with high levels of quantitative workload. In other words, when being confronted with tight deadlines and a huge amount of workload, employees will benefit from the opportunity to decide on the scheduling and pacing of their work. Moreover, the possibility to decide how to accomplish the work task in the face of output pressure will enhance feelings of self-determination and will lead to reduced levels of emotional exhaustion.

On the other hand, job control, as timing and method control, will not prevent impairments of employees’ well-being when coping with emotional dissonance. On the contrary, the current study points out that enhancing job control can even harm employees’ psychological well-being when their jobs are characterized by high levels of emotional dissonance. Furthermore, it is suggested that the adverse effect of high levels of job control may not only manifest in occupations with high emotional dissonance, but also in occupations with other demands on self-control (e.g., resisting distractions, overcoming inner resistances, and impulse control [70]). In these jobs, implementing specific routines and habitual strategies might contribute to employees’ psychological well-being by reducing the amount of effortful choices. For example, by providing precise recommendations on how to structure and schedule a workday, and by recommending some specific methods to accomplish work tasks, the amount of necessary decisions an employee has to make can be reduced. In service occupations, for instance, a standardized complaint management could contribute to reducing job control by providing routine implementations of handling deviant customers. Instead of necessitating ad-hoc decisions on the right strategy to appease an indignant customer, employees can proceed according to an organizationally defined pattern, which necessitates lower amounts of self-control.

Finally, in addition to implementing strategies for managing job control, the well-being of employees could also be improved by addressing quantitative workload and emotional dissonance directly. Nevertheless, changes in the administrative structure that aim to reduce quantitative workload are expected to sacrifice organizational outputs and harm productivity [4]. Similarly, abolishing emotional display rules in service occupations is associated with reduced customer satisfaction [71]. Therefore, the possibilities to reduce these job demands without adversely affecting important organizational outputs are highly limited. Instead, we suggest implementing intervention programs that help employees to deal with job demands. In this context, several promising intervention programs have been shown to be effective. For instance, cognitive-behavioral interventions that train employees in regulating thoughts and emotions have been demonstrated to be more effective with regard to stress reduction than other intervention programs [72,73]. Additionally, prior research has revealed that self-regulation training programs can enhance the ability to execute self-control through repeated self-control exertion [74] and that time management trainings can support employees when facing challenging deadlines [75]. Thus, training programs that are targeted at enhancing the ability to deal with quantitative workload and emotional dissonance might prevent employees to suffer from impaired psychological well-being.

In conclusion, managers and organizations need to consider the advantages and disadvantages of job control and need to understand that identifying the predominant job demand is a prerequisite for deciding on the optimal level of job control. There are specific instances when enhancing job control provides possibilities to cope with job demands, and there are other instances when alternative strategies provide more promising opportunities to enhance employees’ psychological well-being.

## 5. Conclusions

### Is Job Control a Double-Edged Sword?

The present study tested direct relationships and interactions of job demands and job control in a two-wave panel design. By integrating notions of the JD-C model and the strength model of self-control, we shed new light on the ambiguous role of job control. The major contribution of this research is the finding that job control can be a double-edged sword. This result provides useful information to organizations, as well as to researchers, because the adverse consequences of job control have been overlooked in research and practice so far. Understanding the complexities of job control and the circumstances, which jeopardize the employees’ psychological well-being, is a first step in attempting to reduce the potential adverse effects of job control.

## Figures and Tables

**Table 1:** Means, standard deviations, internal consistencies (*Cronbach’s Alpha*) and bivariate correlations of study variables.

Variable		Time	1	2	3	4	5	6	7	8
1	Quantitative workload	T1	(*0.82*)							
2		T2	**0.66**	(*0.80*)						
3	Emotional dissonance	T1	**0.18**	**0.17**	(*0.94*)					
4		T2	0.12	**0.18**	**0.79**	(*0.95*)				
5	Job control	T1	**−0.33**	**−0.28**	**−0.35**	**−0.32**	(*0.85*)			
6		T2	**−0.23**	**−0.26**	**−0.34**	**−0.40**	**0.70**	(*0.86*)		
7	Emotional exhaustion	T1	**0.42**	**0.38**	**0.49**	**0.48**	**−0.45**	**−0.37**	(*0.91*)	
8		T2	**0.32**	**0.47**	**0.49**	**0.56**	**−0.25**	**−0.34**	**0.74**	(*0.91*)
	*M*		2.62	2.61	3.52	3.57	3.23	3.24	2.19	2.27
	*SD*		0.93	0.84	0.97	0.99	0.59	0.56	0.92	0.93

Note: *N* = 139; numbers in italics are internal consistencies (*Cronbach’s Alpha)*; numbers in bold are significant at *p* < 0.05.

**Table 2:** Model comparison.

#	Model	*χ*^2^	df	CFI	TLI	RMSEA	Model Comparison	Δ*χ*^2^	Δdf
1	Stability Model	27.42	*12*	0.973	0.937	0.096			
2	Causality Model	11.46	9	0.996	0.987	0.044	1 vs. 2	15.96 **	3
3	Reversed Causation Model	18.80	9	0.983	0.947	0.089	1 vs. 3	8.62 *	3
4	Reciprocal Model	4.17	6	1.000	1.000	0.000	1 vs. 4	23.25 **	6
							2 vs. 3	−7.34	0
							2 vs. 4	7.29	3
							3 vs. 4	14.63 **	3

Note: *N* = 139; * *p* < 0.05; ** *p* < 0.01.

**Table 3:** Parameter estimates of path models.

	Model 2:		Model 5:	
	*Causality Model*		*Moderation Model*	
	*γ*	SE	*γ*	SE
Autoregressive Effects				
Quantitative workload	0.55 **	0.05	0.55 **	0.05
Emotional dissonance	0.76 **	0.05	0.76 **	0.05
Job control	0.38 **	0.03	0.38 **	0.03
Emotional exhaustion	0.57 **	0.06	0.56 **	0.06
Predicting Emotional Exhaustion T2				
Quantitative workload (T1)	0.07	0.05	0.08	0.05
Emotional dissonance (T1)	0.18 **	0.05	0.17 **	0.05
Job control (T1)	0.14 **	0.05	0.09	0.06
Quantitative workload × job control (T1)			−0.09 *	0.05
Emotional dissonance × job control (T1)			0.11 **	0.04

Note: *N* = 139; * *p* < 0.05; ** *p* < 0.01.
